# Genome-wide identification of the nuclear redox protein gene family revealed its potential role in drought stress tolerance in rice

**DOI:** 10.3389/fpls.2025.1562718

**Published:** 2025-04-22

**Authors:** Zijie Liu, Xingfei Zheng, Dabing Yang, Lanzhi Li, Hexing Yin

**Affiliations:** ^1^ Hunan Engineering & Technology Research Center for Agricultural Big Data Analysis & Decision-Making, College of Plant Protection, Hunan Agricultural University, Changsha, China; ^2^ Hubei Key Laboratory of Food Crop Germplasm and Genetic Improvement, Food Crop Institute, Hubei Academy of Agricultural Sciences, Wuhan, China; ^3^ Crop Phenomics Research Center, Huazhi Biotechnology Co., Ltd, Changsha, China

**Keywords:** rice, nucleoredoxin gene family, drought stress, genome-wide identification, expression pattern

## Abstract

**Introduction:**

Thioredoxins (TRX) are redox-active proteins critical for plant stress adaptation. As a TRX family member, nucleoredoxin (NRX) maintains drought-induced redox homeostasis, yet its genome-wide characterization in rice remains uninvestigated.

**Methods:**

Using HMMER3.0 (E-value <1e-5) and TBtools, we identified OsNRX genes across three rice varieties (Minghui63, Nipponbare, 9311). Conserved domains were verified by SMART/CDD, while promoter cis-elements were systematically predicted with PlantCARE. Tissue-specific expression patterns were analyzed using RiceXPro data, and drought responses were quantified via qRT-PCR in drought-tolerant (Jiangnong Zao 1B) versus sensitive (TAISEN GLUTINOUS YU 1157) varieties under PEG6000 stress.

**Results:**

Ten OsNRX genes were classified into three subfamilies (NRX1/NRX2/NRX3) exhibiting conserved domain architectures. Promoter analysis identified abundant stress-responsive elements (ABRE, MBS) and phytohormone signals (ABA/JA/SA). Tissue-specific expression profiles revealed NRX1a dominance in roots/hulls, versus NRX1b/NRX2 enrichment in endosperm. Drought stress triggered rapid OsNRX upregulation (20-53-fold within 3-6h), with tolerant varieties showing earlier NRX2 activation than sensitive counterparts.

**Discussion:**

OsNRX genes exhibit dynamic drought-responsive regulation, while their spatiotemporal expression in glumes, embryos, and endosperm suggests potential dual roles in stress adaptation and grain development. These results provide molecular targets for improving drought resilience in rice breeding.

## Introduction

Nucleoredoxin (NRX), initially discovered by Kurooka et al. (1997) in the nucleus of mouse (*Mus musculus*) cells, is named for its ability to reduce oxygen and is classified within the thioredoxin (TRX) superfamily due to its TRX-like domain. The TRX superfamily is involved in a series of physiological and biochemical processes within the cell that are associated with redox reactions, by reducing disulfide bonds in their target proteins (Piaz et al., 2010). Laughner et al. (1998) were the first to isolate plant nucleoredoxin from maize (*Zea mays*), confirming that NRX proteins possess significant insulin-degrading activity and the capacity to catalyze redox reactions. Marchal et al. (2014) categorized NRX into three subgroups based on the number of TRX-like domains: Type I NRX contains three TRX-like domains, with the first and third domains harboring typical WC[G/P]PC redox sites, while the second domain is an atypical TRX domain with weaker activity (Cheng et al., 2021a); Type II NRX contains two TRX domains with atypical active sites WY[P/A][K/P]C and [W/R/H]C[L/A/V/R]P[C/G]; Type III NRX contains three TRX domains, with the first domain having a highly conserved WCRPC redox site, and the second domain typically containing a classic Trx active site WCPP[C/F/S].

NRX plays a crucial role in plants’ response to drought stress. Zhang et al. (2021) constructed overexpression and RNA interference wheat lines for *TaNRX1* to investigate its mechanism in regulating wheat drought tolerance. The results indicated that the overexpression lines had higher drought tolerance indicators such as survival rate, leaf chlorophyll, proline, soluble sugar content, and antioxidant enzyme activity compared to the wild type, while the RNAi lines showed the opposite, demonstrating that *TaNRX1* positively regulates wheat’s tolerance to drought stress. Tian et al. (2019) demonstrated that *TaNRX1* interacts with *TaPDI*, *TaTRX-h*, and *TaPP2Ac* proteins in the nucleus and cell membrane, and that the overexpression of *TaPDI*, *TaTRX-h*, and *TaPP2Ac* alleviated the inhibitory effects of drought stress on yeast growth. In addition, Zhang et al. (2022) showed that overexpression of SiNRX1 in foxtail millet (*Setaria italica*) enhanced Arabidopsis tolerance to drought and salt stress. Jedmowski et al. (2014) conducted drought stress experiments on two sorghum (*Sorghum bicolor*) varieties with differing drought tolerance and found that SbNRX expression significantly increased during both stress and recovery phases in the drought-tolerant variety, whereas in the drought-sensitive variety, SbNRX expression increased only during the stress phase.

NRX is also involved in regulating plant growth and development and responding to other stresses. In rice (*Oryza sativa*), *NRX* regulates cytokinin signaling by inhibiting the dimerization of the *Oryza sativa* histidine kinase 4 (*OHK4*), thereby influencing regeneration capacity and leaf senescence (Yao et al., 2023). In *Arabidopsis*, *AtNRX1* has been shown to integrate signals from maternal tissues and guide pollen tube growth toward the ovule, thereby regulating pollen tube growth and pollen fertility (Qin et al., 2009). *AtNRX2* is specifically induced by jasmonic acid (JA) and promotes trichome formation by regulating the expression of genes related to trichome formation (Lee et al., 2020). In tomato (*Lycopersicon esculentum*), *SLNRX1* is a positive regulator of heat stress tolerance, protecting cells from oxidative damage and protein denaturation induced by heat stress by enhancing antioxidant capacity (Cha et al., 2022). In grape (*Vitis vinifera*), Gauthier et al. (2014) found that after inoculation with grape downy mildew *Plasmopara viticola*, the expression of *NRX1* was specifically induced by salicylic acid (SA), thereby improving grape resistance to downy mildew.

Rice is a major staple crop worldwide and serves as the primary energy source for more than half of the global population (Hassan et al., 2023). Due to its high water consumption during production, rice yield is highly susceptible to drought (Panda et al., 2021). To date, studies on the *NRX* gene family have primarily been derived from research on the *TRX* family, but a systematic investigation of the *NRX* gene family in rice remains lacking. Therefore, this study aims to identify the *OsNRX* gene family and investigate its role in rice’s response to drought stress, providing a foundation for in-depth functional analysis of rice *NRX* genes and offering scientific support for molecular breeding to enhance drought resistance in rice. Using bioinformatics methods, this study identified the *NRX* genes in the rice genome and conducted a comprehensive analysis of their protein physicochemical properties, subcellular localization, protein structure, conserved domains, phylogenetic tree and cis-acting elements. Furthermore, the expression patterns of the *OsNRX* gene family under drought stress were examined to elucidate the regulatory effects of drought stress on *OsNRX* gene expression.

## Materials and methods

### Data sources

Genomic data and annotation files for the rice varieties *Nipponbare* and *9311* were downloaded from the Ensembl Plants database (http://plants.ensembl.org/info/data/ftp/index.html) (Yates et al., 2022), accessed on 2023.7.27. The whole-genome data and its annotation files for the rice variety *Minghui63* were obtained from the Rice Information Network RIGW (http://rice.hzau.edu.cn/cgi-bin/rice_rs1/download_ext) (Song et al., 2018), accessed on 2023.7.27. The HMM file for the *NRX* gene (PF13905) was downloaded from the InterPro database (https://www.ebi.ac.uk/interpro) (Paysan-Lafosse et al., 2023), accessed on 2023.7.27. The genotype data used for SNP Analysis were obtained from Meng et al. (2022).

### Identification of *OsNRX* gene family members

The hmmsearch program of HMMER3.0 and the Blast Compare Two Seqs program of TBtools are employed to search for proteins similar to the NRX domain within the total protein sequences of rice (Eddy, 2011; Chen et al., 2020), with an E-value of 1×10^-5^ used to filter the search results, yielding candidate rice *NRX* gene family members. The intersection of the two results is the final set of candidate gene family members. The protein sequences of the final set of candidate gene family members were submitted to the SMART (http://smart.embl.de) (Letunic et al., 2021) and CDD (https://www.ncbi.nlm.nih.gov/cdd) (Wang et al., 2023) databases, and sequences containing the NRX domain were confirmed as rice *NRX* gene family members.

### Physicochemical property analysis and subcellular localization of *OsNRX* proteins

The online software ExPASy (http://web.expasy.org/protparam) (Duvaud et al., 2021) was utilized to analyze the protein sequences encoded by the rice *NRX* genes, predicting their relative molecular weight, isoelectric point, instability index, grand average of hydropathy, and hydrophobicity. The online tool Plant-mPLoc (http://www.csbio.sjtu.edu.cn/bioinf/plant-multi) (Chou and Shen, 2010) was used for subcellular localization prediction.

### Secondary and 3D structure prediction of *OsNRX* proteins

The SOPMA website (https://npsa-pbil.ibcp.fr/cgi-bin/npsa_automat.pl?page=npsa_sopma.html) (Geourjon and Deléage, 1995) was used to predict the secondary structure of proteins encoded by the rice *NRX* gene family; the SWISS-MODEL website (https://swissmodel.expasy.org/interactive) (Waterhouse et al., 2018) was used for 3D structure prediction of the *OsNRX* protein family.

### Conserved domain and motif analysis of *OsNRX* proteins

TBTools and the online software MEME (https://meme-suite.org/meme/tools/meme) (Bailey et al., 2015) were used to predict conserved domains and motifs of the *OsNRX* proteins. MUSCLE in MEGAX was used to align the rice NRX protein sequences to obtain the evolutionary relationships between NRX proteins (Kumar et al., 2018). MEME was used to identify conserved motifs (Motif) in the NRX protein sequences, with a maximum of 10 conserved structural domains and motif width set to 6-20 bp. TBtools were then used to analyze and visualize the conserved motif information.

### Phylogenetic analysis of the *OsNRX* family

Genomic data and annotation files for wheat, foxtail millet, Arabidopsis, soybean, and maize were downloaded from the Ensembl Plants database (http://plants.ensembl.org/info/data/ftp/index.html), accessed on 2023.8.2. The method described above for identifying rice *NRX* gene family members was used to identify *NRX* gene family members in wheat, foxtail millet, Arabidopsis, soybean, and maize. ClustalW in MEGAX was used to align the NRX protein sequences of these species with the rice NRX protein sequences, and a phylogenetic tree was constructed using the Neighbor-Joining (NJ) method with a bootstrap value set to 1000 and other parameters default. The iTOL website (https://itol.embl.de) (Letunic and Bork, 2024) was used to beautify the phylogenetic tree.

### Prediction of cis-acting elements in the *OsNRX* family

To identify cis-acting elements related to the *NRX* genes, 2000 bp of genomic sequence upstream of the transcription start site were extracted and submitted to the PlantCARE website (http://bioinformatics.psb.ugent.be/webtools/plantcare/html) (Lescot, 2002) for prediction of cis-acting elements. Common cis-acting elements, light response elements, and elements of unknown function were removed, and a diagram of the cis-acting elements of the *OsNRX* family was drawn.

### Expression analysis of the *OsNRX* family at different developmental stages

The expression data of *OsNRX* genes across various tissues and developmental stages of *Nipponbare* rice were downloaded from the RiceXPro V3 database (https://ricexpro.dna.afrc.go.jp/index.html) (Sato et al., 2013). The data were processed as follows: (1) Cy3 raw signals were normalized to the 75th percentile using Agilent Feature Extraction software (version 9.5.3.1); (2) log2 transformation was applied. The analyzed tissues included leaf, sheath, root, stem, panicle, anther, pistil, lemma, palea, ovary, embryo, and endosperm, while the developmental stages covered the vegetative, reproductive, and mature phases. The R heatmap package was used to visualize the expression patterns of *OsNRX* genes across these tissues and developmental stages.

### Plant materials and stress treatments

In this study, we used two varieties obtained from the Institute of Grain Crops, Hubei Academy of Agricultural Sciences: *Jiangnong Zao 1B*, which is characterized by drought resistance during the seedling stage, and *TAISEN GLUTINOUS YU 1157*, which is known for its drought sensitivity at the seedling stage. Cultivation was conducted in an artificial climate chamber (16 h of light at 28°C, 8 h of darkness at 26°C, 70% relative humidity, and light intensity of 200 μmol/m^2^/s). Cultivate the plants with tap water for the first 4 days, then with half-strength Yoshida nutrient solution (Coolaber, Beijing, China) for the next 3 days, followed by full-strength Yoshida nutrient solution for 7 days, changing the solution every two days. At 14 days old, subject the seedlings to drought stress using full-strength Yoshida nutrient solution supplemented with 20% PEG6000. Samples were collected at 0, 3, 6, 12, and 24 h post-stress. For each species, three plants with uniform growth conditions were selected, and 0.1 g of fresh young leaves were quickly placed in 1.5 mL centrifuge tubes, tightly capped, and stored in liquid nitrogen and then stored in an ultra-low temperature refrigerator at -80°C.

### Real-time quantitative PCR (qRT-PCR)

Fluorescence quantitative PCR primers were designed using Primer Premier 6 (**Supplementary Table S5**), with *OsACT* selected as the reference gene. The melting temperature is 55-60°C, the primer length is 19-23bp, the GC content is 47-57%, and the amplicon size is 179-222bp. The total RNA of the sample was extracted using the RNA Easy Fast Plant Tissue Kit (Tiangen, China). cDNA was synthesized using RevertAid Reverse Transcriptase (Thermo Fisher Scientific, USA; Cat# EP0441), following the manufacturer’s protocol. qRT-PCR was then performed using the PerfectStart Green qPCR SuperMix (TransGen Biotech, China) on a QuantStudio 1 Plus Real-Time PCR System (Thermo Fisher Scientific, USA). Related experimental parameters are provided in **Supplementary Table S6**.

### Statistical analysis

Each sample was repeated for 3 times, and the data were processed by 2^-△△Ct^ method (Livak and Schmittgen, 2001). Statistical analysis was performed using R (R Core Team, 2024). Data were analyzed using the Student T-test, and least significant difference *post hoc* tests were used to determine significant differences between means (*, statistically significant at P < 0.1; **, statistically significant at P < 0.05; ***, statistically significant at P < 0.01).

### SNP analysis

The reference genome used for SNP calling was the Os-Nipponbare-Reference-IRGSP-1.0 (Li and Durbin, 2010). Variants were identified using the GATK (DePristo et al., 2011), with single-nucleotide polymorphisms (SNPs) retained if they met the following criteria: minor allele frequency (MAF) ≥ 5% and missing rate ≤ 20%. Missing genotypes were imputed using IMPUTE2 (Howie et al., 2009), and SNPs specific to *Nipponbare*, *Minghui63*, *9311*, *Jiangnong Zao 1B*, and *TAISEN GLUTINOUS YU* were extracted using Plink (Purcell et al., 2007).

## Results

### Identification and basic characteristics of *OsNRX* gene family members

**Figure 1:**
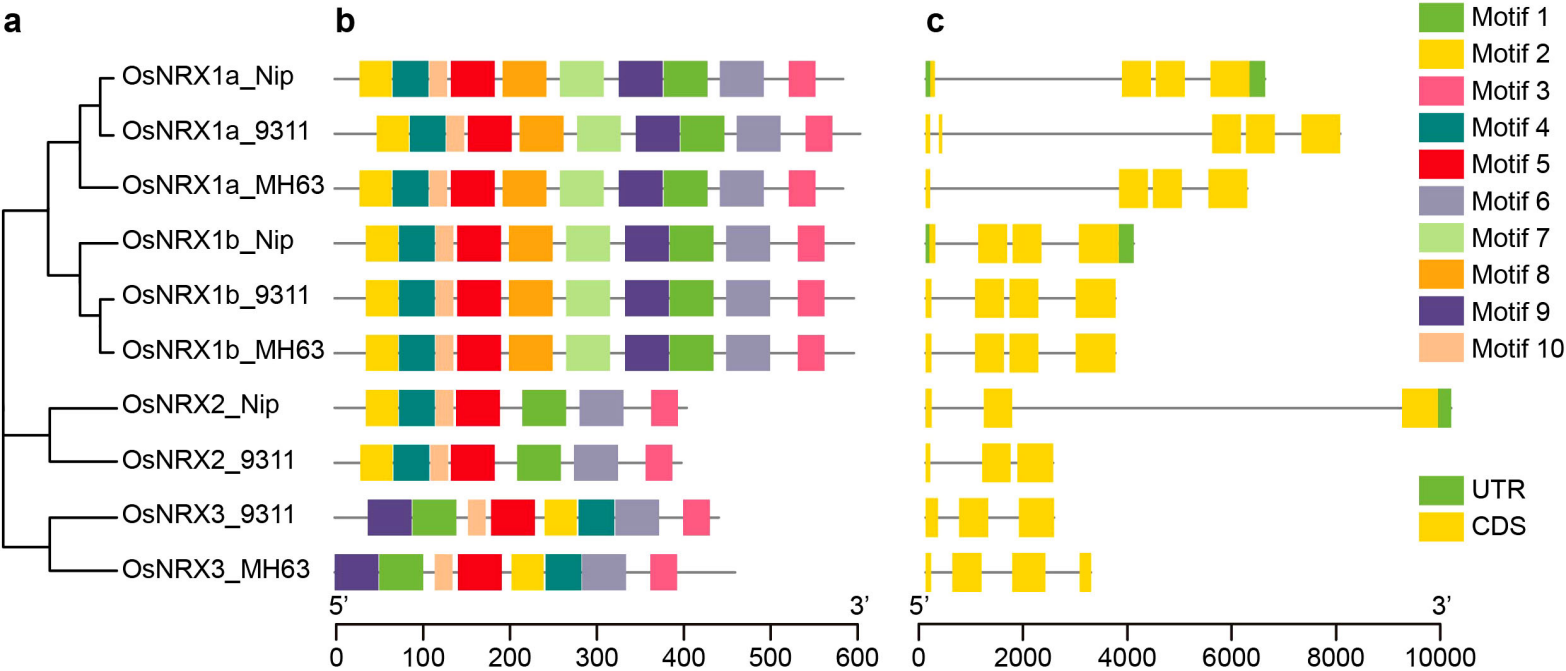
Phylogenetic relationship, conserved motif structure and gene structure of OsNRX protein. **(a)** The phylogenetic tree of *OsNRX*. MH63 represented *Minghui63*(*indica*), Nip represented *Nipponbare*(*japonica*), 9311 represented *9311*(*indica*). **(b)** The distribution of conserved motifs. Boxes with different colors represented different conserved motifs (Motif 1-10). **(c)** The gene structure of *OsNRX*. Green boxes represented the UTRs, yellow boxes represented exons.

A total of 10 candidate *NRX* genes were identified in the rice varieties *Minghui63* (indica), *Nipponbare* (japonica), and *9311* (indica) using HMMER3.0 and TBtools software (**Supplementary Table S1**). The protein sequences of these candidate gene family members were analyzed using SMART and CDD, and the results showed that all candidates contained the NRX domain. Following the classification method of *NRX* family by Marchal et al. (2014) (**Figure 1a**), the *NRX* genes in *Minghui63* were named *OsNRX1a_MH63*, *OsNRX1b_MH63*, and *OsNRX3_MH63*; in *Nipponbare*, they were named *OsNRX1a_Nip*, *OsNRX1b_Nip*, and *OsNRX2_Nip*; and in *9311*, they were named *OsNRX1a_9311*, *OsNRX1b_9311*, *OsNRX2_9311*, and *OsNRX3_9311*.

The physicochemical properties and subcellular localization of the 10 NRX protein sequences in rice were analyzed using the online software ExPASy and Plant-mPLoc, predicting their isoelectric point, molecular weight, grand average of hydropathicity (**Table 1**). The size of OsNRX proteins ranges from 388 to 581 amino acids; their molecular weights range from 43.10 to 65.36 kDa; and their isoelectric points are all below 7, indicating acidic properties; the average hydrophilicity index is less than 0, suggesting that the *NRX* gene family proteins are hydrophilic. Subcellular localization predictions suggest that *OsNRX* family members may be distributed across different organelles, such as the nucleus, chloroplast, or cytoplasm. These details imply that *OsNRX* members may perform distinct functions in different parts of the cell.

**Table 1:** Basic characteristics of *OsNRX* gene family members.

Gene name	Gene ID	Chromosome location	Number of amino acid(aa)	Isoelectric point	Molecular weight (kDa)	Grand average of hydropathicity	Predicted subcellular localization
*OsNRX1a_MH63*	MH03g0321000	Chr3:16314247-16319663	569	4.92	63.24	-0.32	Nucleus, chloroplast
*OsNRX1b_MH63*	MH03g0321400	Chr3:16355718-16358916	581	4.82	64.45	-0.28	Nucleus
*OsNRX3_MH63*	MH04g0621000	Chr4:27807008-27809640	430	6.25	48.94	-0.30	Chloroplast
*OsNRX1a_Nip*	Os03g0405500	Chr3:16590192-16595911	569	4.95	63.29	-0.03	Nucleus, chloroplast, cytoplasm
*OsNRX1b_Nip*	Os03g0405900	Chr3:16628549-16632054	581	4.82	64.42	-0.28	Nucleus, cytoplasm
*OsNRX2_Nip*	Os01g0794400	Chr1:33642080-33650929	394	5.03	43.78	-0.30	Nucleus, chloroplast
*OsNRX1a_9311*	BGIOSGA012836	Chr3:18471613-18478597	588	5.02	65.36	-0.30	Nucleus, chloroplast
*OsNRX1b_9311*	BGIOSGA012837	Chr3:18517506-18520704	581	4.82	64.45	-0.28	Nucleus
*OsNRX2_9311*	BGIOSGA004598	Chr1:37053808-37055959	388	5.06	43.10	-0.28	Nucleus, cytoplasm
*OsNRX3_9311*	BGIOSGA014382	Chr4:29656770-29659559	448	5.96	51.01	-0.40	Cytoplasm

MH63 represented *Minghui63*(indica), Nip represented *Nipponbare*(japonica), 9311 represented *9311*(indica).

### Gene structure analysis and protein conserved motif analysis of *OsNRX*

To explore the sequence information of the *OsNRX* gene family members, the gene structure and conserved motifs were analyzed using MEME and TBtools software. Regarding the protein conserved motifs, the number and distribution of motifs within the *OsNRX* subfamily members were largely consistent (**Figure 1b**). All *OsNRX* family members contain Motif1, Motif2, Motif3, and Motif6. Notably, Motif1 and Motif2 harbor the characteristic “WCGPC” sequence and are located within the TRX domain, whereas Motif3 and Motif6 are found in the cysteine-rich C-terminal domain, the function of which remains unclear. The gene structures of *OsNRX* genes show some variation (**Figure 1c**), with *OsNRX2-Nip* being the longest at 8.8 kb and *OsNRX3_MH63* the shortest at only 2.1 kb. All *OsNRX* genes contained at least three CDS segments, with only *OsNRX1a-Nip*, *OsNRX1b-Nip*, and *OsNRX2-Nip* containing UTR fragments.

### Secondary and 3D structure prediction of *OsNRX* proteins

**Figure 2:**
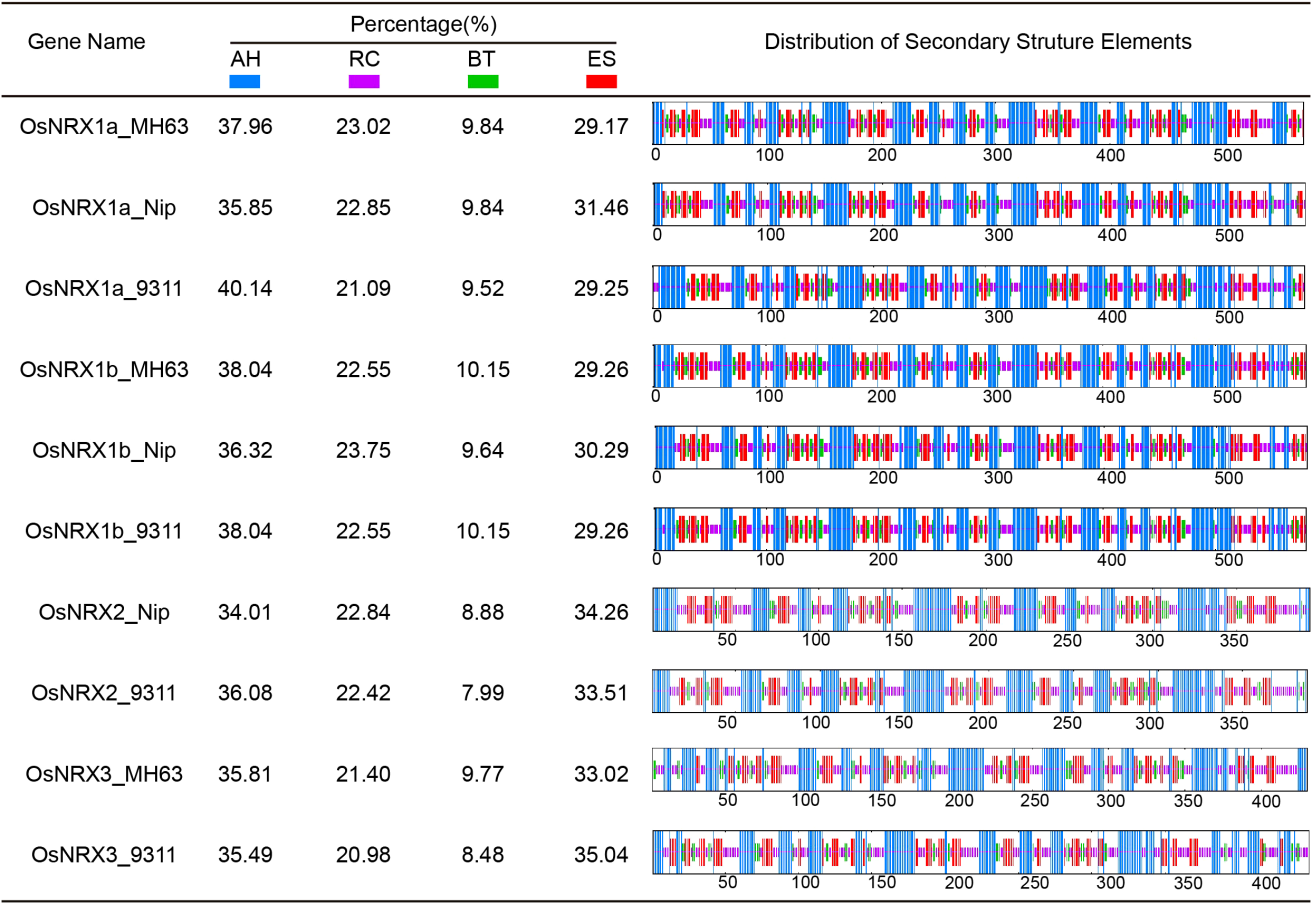
Secondary structure analysis of *OsNRX* proteins. MH63 represented *Minghui63*(*indica*), Nip represented *Nipponbare*(*japonica*), 9311 represented *9311*(*indica*). The blue color represents alpha helix (AH), the purple color represents random coil (RC), the green color represents beta turn (BT) and the red color represents extended strand (ES).

Secondary structures of *OsNRX* proteins were analyzed by the SOPMA online website.*OsNRX1a OsNRX1b* and *OsNRX2* proteins have similar composition and structure in three rice varieties, while the *OsNRX3* protein shows some variation among different varieties (**Figure 2**). All *OsNRX* proteins were consisted of four secondary structures: alpha helix, extended helix, random coil, and extended strand, and the proportions of alpha helix > extended strand > random coil> beta turn.

**Figure 3:**
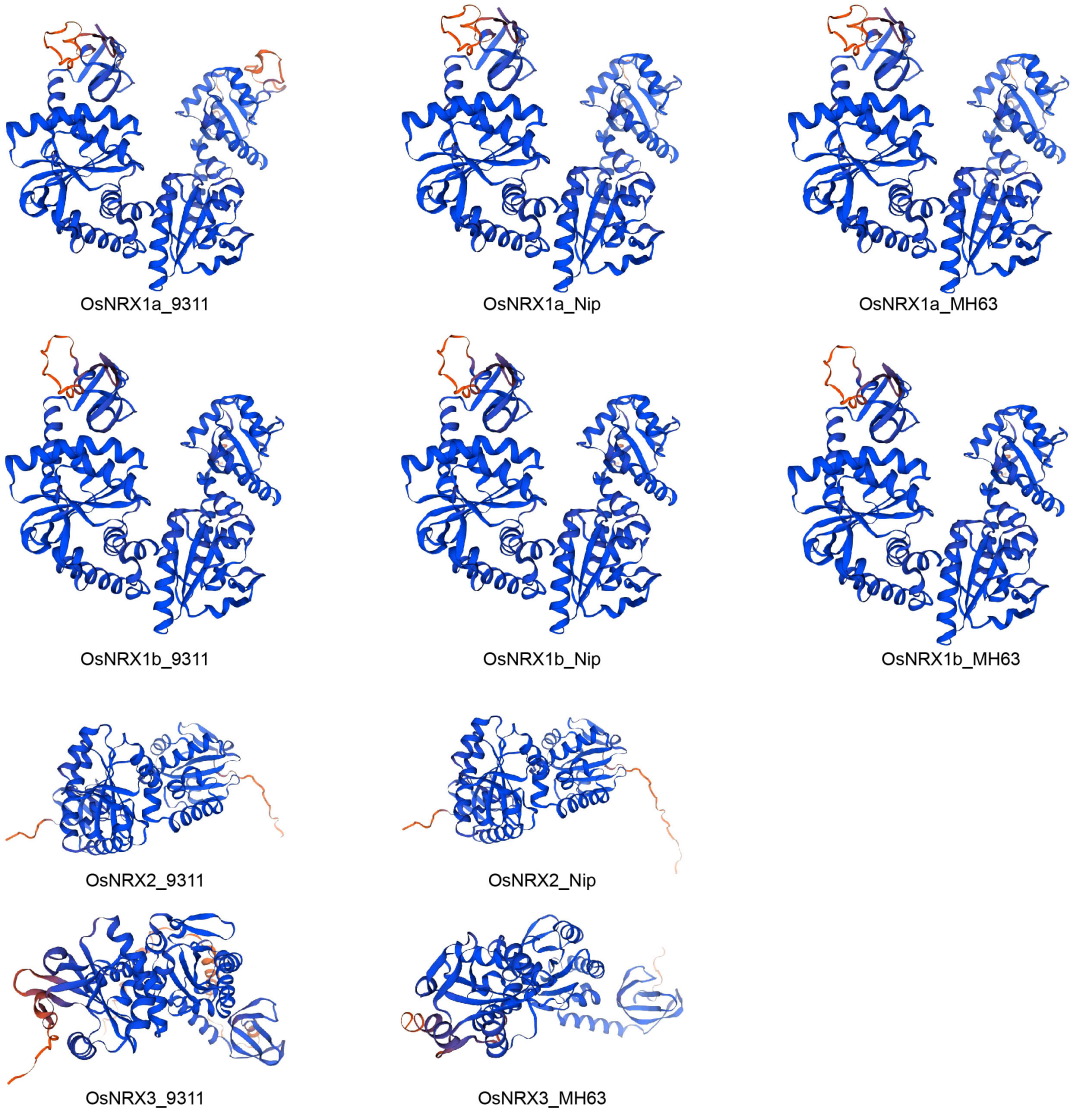
Protein 3D structure prediction model of *OsNRX* gene families. MH63 represented *Minghui63*(*indica*), Nip represented *Nipponbare*(*japonica*), 9311 represented *9311*(*indica*).

The 3D structures of proteins encoded by the OsNRX gene family were predicted using the online SWISS-MODEL platform. The results revealed that the 3D structures of NRX1a and NRX1b are highly similar across the three rice varieties. Similarly, the tertiary structures of NRX2 show high similarity between Nipponbare and 9311, whereas the 3D structures of NRX3 exhibit lower similarity between Minghui 63 and 9311 (**Figure 3**). The GMQE values for all these predicted structures exceed 0.8, indicating relatively high reliability of the predictions (Biasini et al., 2014).

### Phylogenetic tree analysis of the Os*NRX* gene family

**Figure 4:**
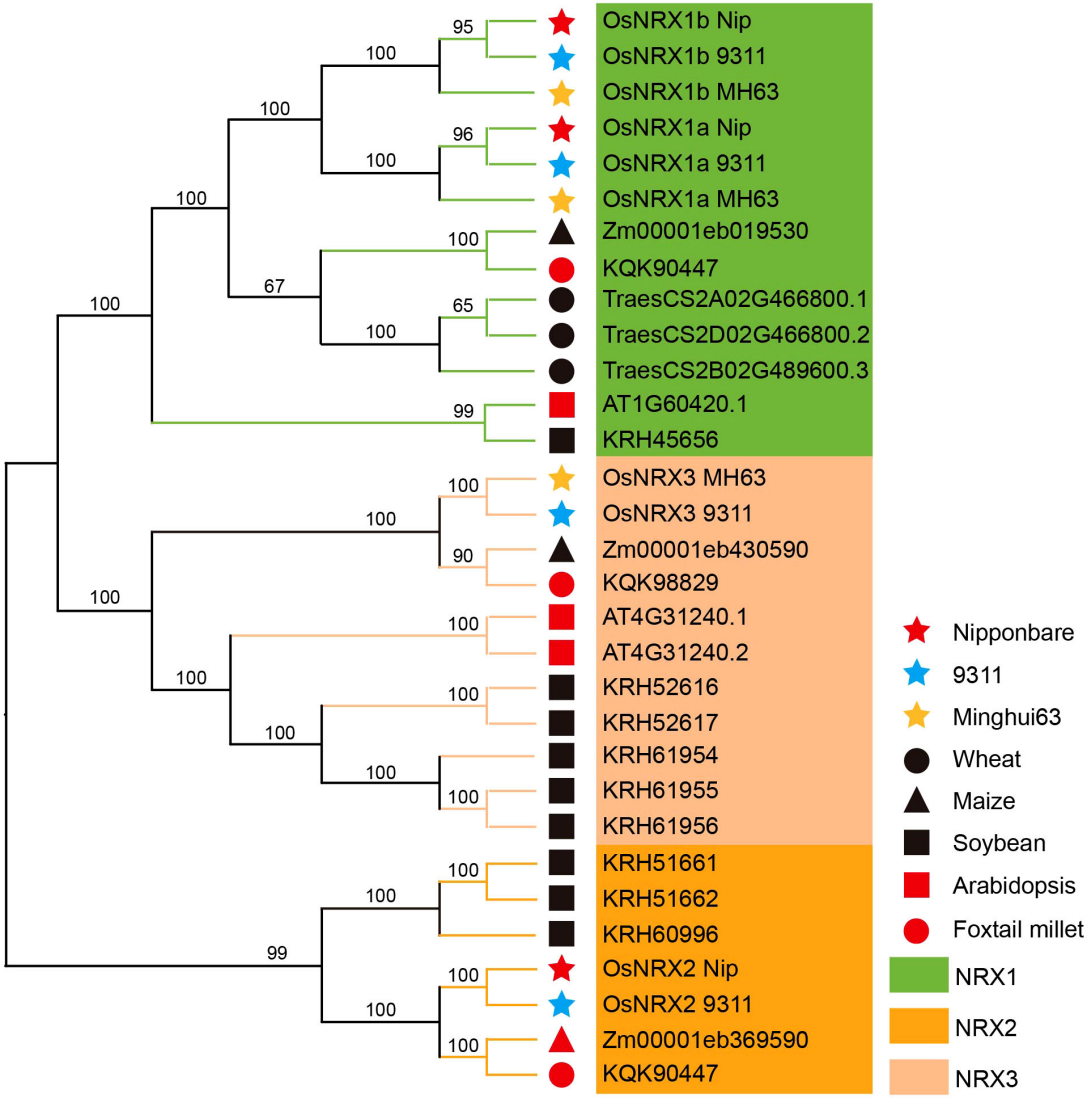
Phylogenetic tree of *NRX* gene family proteins in rice and five species. Phylogenetic trees were constructed using the Neighbor-Joining (NJ) method of the MEGAX software with the bootstrap value set to 1000.

To investigate the evolutionary relationships of the OsNRX genes, NRX gene family members were identified in wheat, foxtail millet, Arabidopsis, soybean, and maize. The phylogenetic tree of these *NRX* family members was constructed (**Figure 4**, **Supplementary Table S1**). This comprehensive analysis encompasses a diverse range of species, such as rice with three distinct varieties—*Minghui63*, *Nipponbare*, and *9311*—as well as wheat, foxtail millet, Arabidopsis, soybean, and maize. The phylogenetic tree of *NRX* gene members is divided into three subfamilies, with 12 genes assigned to the *NRX1* subfamily, 10 genes to the *NRX2* subfamily, and 6 genes to the *NRX3* subfamily. In conjunction with the protein sequences of the *NRX* genes, the results of the phylogenetic tree are fully consistent with the *NRX* subfamily classification by Marchal et al. (2014). Consistent with their evolutionary relationships, the NRX genes of the three rice varieties show the closest phylogenetic relationships, followed by wheat, foxtail millet and maize, while exhibiting the most distant relationships with Arabidopsis and soybean. All 6 species have at least one NRX1 gene, and the three rice varieties each contain at least two NRX1 genes. The study by Hazra et al. (2024) demonstrated that NRX1 genes are present in 15 species, and that *Oryza sativa*, *Populus trichocarpa*, and *Vitis vinifera* each contain multiple *NRX1* members. Our findings are consistent with their results, further suggesting that *NRX1* is a conserved gene that may play a critical role in plants. The *NRX1* genes of wheat, foxtail millet and maizet are more closely related to those of rice in the phylogenetic tree. NRX1 in foxtail millet was able to balances the excess reactive oxygen species (ROS) due to drought and salt stress, reduces oxidative damage in plants, and maintains normal growth and development (Zhang et al., 2022). Combined with the involvement of the *TaNRX1-D* gene in regulating the response to drought stress in wheat (Cheng et al., 2021b), we speculate that the *NRX* family in rice may have similar functions.

### Cis-acting elements of the *OsNRX* family

**Figure 5:**
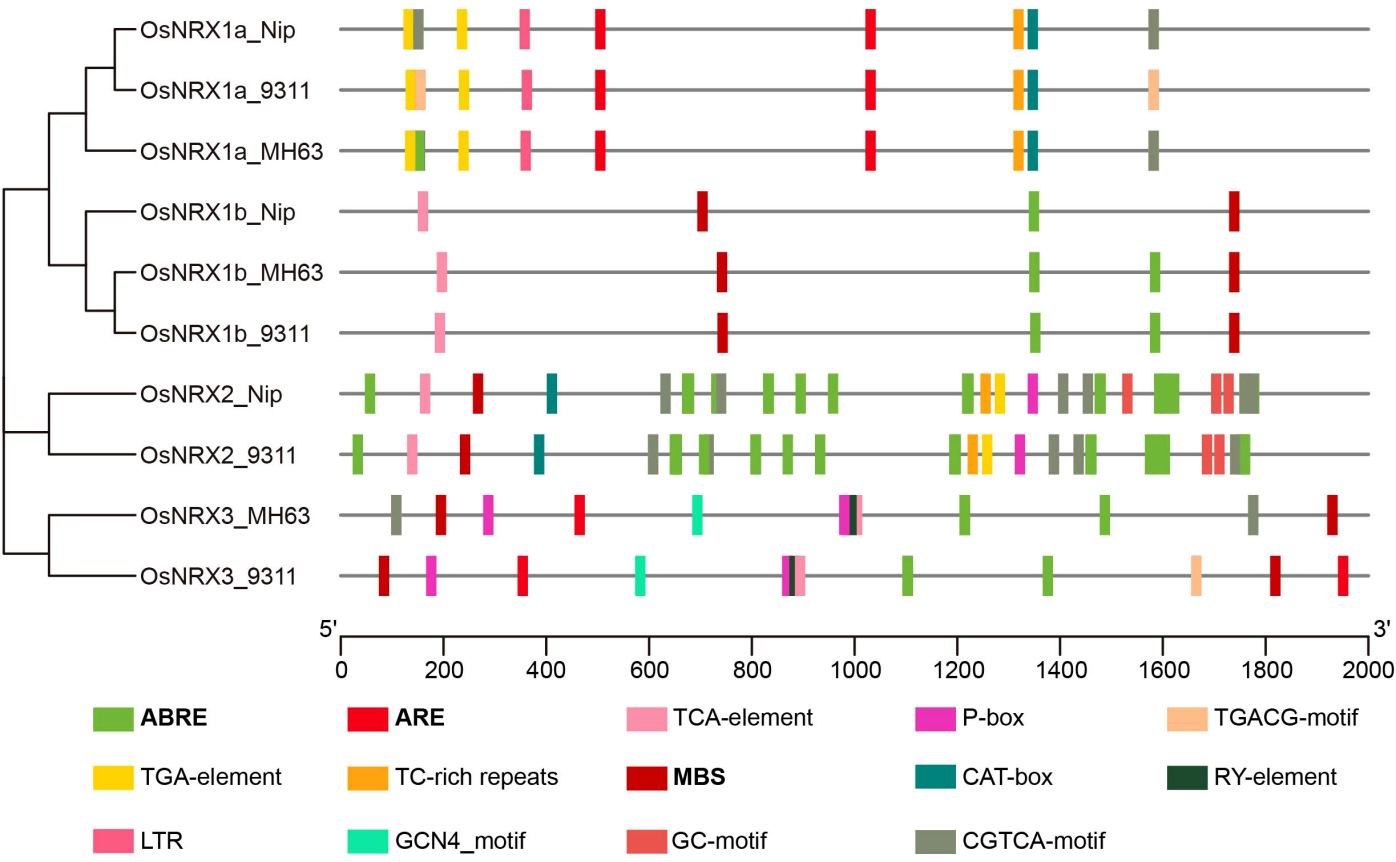
Distribution of cis-acting elements in promoter regions of *OsNRX* genes. MH63 represented *Minghui63* (*indica*), Nip represented *Nipponbare* (*japonica*), 9311 represented *9311* (*indica*). Boxes with different colors represent cis-regulatory elements, with bold fonts indicating cis-regulatory elements related to drought induction, and the scale at the bottom represents the length of the promoter sequence.

To predict the biological functions of the *OsNRX* genes, we conducted a cis-acting element analysis on the 2000 bp promoter region upstream of the start codon of the *OsNRX* genes using the PlantCARE database (**Figure 5**, **Supplementary Table S2**). The results indicated that the promoter regions of *OsNRX*-related genes contain a rich array of cis-acting elements that may significantly influence their biological functions. After initial screening, a variety of cis-acting elements were discovered, some of which are associated with known biological functions, while others are of unknown function. After removing elements of unknown function, a total of 36 different types of cis-acting elements were predicted, summing up to 877 elements. The number and types of cis-acting elements in the promoter region of *OsNRX* gene are obviously different. The analysis revealed significant differences in the number and types of cis-acting elements in the promoter regions of different *OsNRX* genes (**Figure 5**). In terms of quantity, *OsNRX1* has the fewest cis-acting elements, with an average of 9 elements per promoter region; *OsNRX2* has the most, with an average of 45 elements per promoter region; and *OsNRX3* has an average of 18 elements. In terms of types, in addition to common cis-acting elements such as CAAT-box, TATA-box, and light response elements, specific cis-acting elements related to plant response to environmental stress and growth and development were also identified. Among these, abscisic acid response elements (ABRE) were the most abundant, accounting for about 45% of the uncommon cis-acting elements. Additionally, the *OsNRX* family includes elements responsive to JA (CGTCA-motif, TGACG-motif), SA (TCA-element), IAA (TGA-element), GA (P-box), growth and development (CAT-box, GCN4_motif, RY-element), and drought induction (ARE, MBS). The presence of these elements suggests that *OsNRX* genes may be involved in the response and adaptation mechanisms to drought stress by regulating plant water absorption and utilization.

### Expression patterns of the *OsNRX* family at different developmental stages

**Figure 6:**
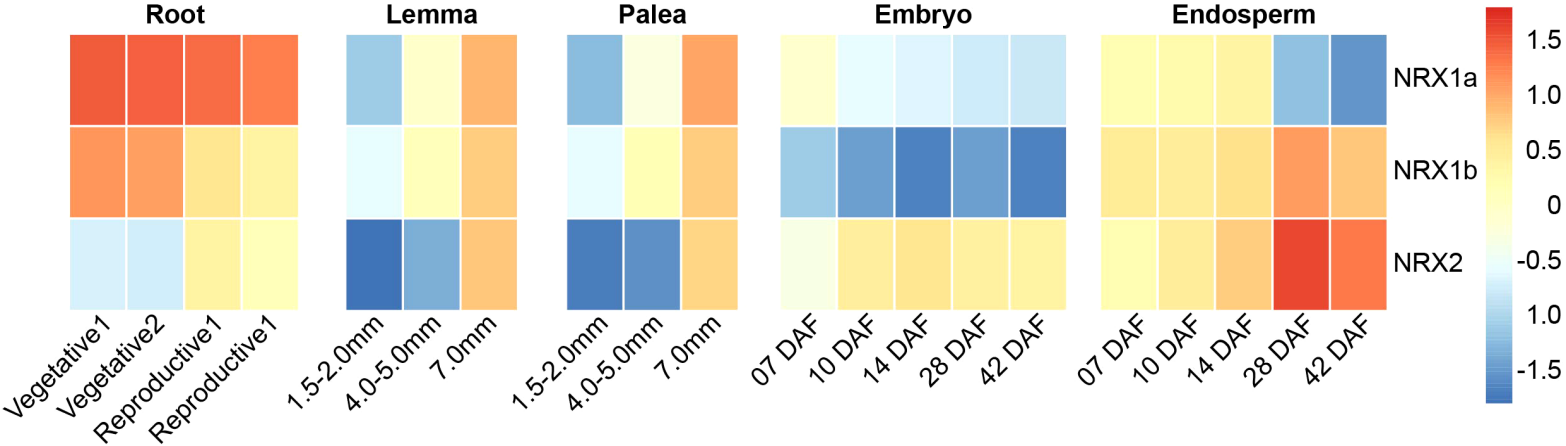
Expression patterns of the *OsNRX* gene family in rice. The color scale represents the relative expression levels, ranging from low (blue) to high (red). “mm” represents millimeters, and “DAF” represents days after flowering.

Both phylogenetic tree analysis and cis-acting element prediction suggested that the OsNRX gene family might be involved in drought stress response. To further investigate the function of OsNRX genes at different developmental stages and the possibility of their involvement in response to drought stress, we downloaded gene expression data from the RiceXpro database (https://ricexpro.dna.afrc.go.jp/index.html) for different tissues of *Nipponbare* at vegetative and reproductive growth stages. The RiceXpro database employs a single microarray system for data collection, ensuring consistency across experiments and providing a reliable platform for comparing gene expression across tissues/organs, growth stages, and experimental conditions (Sato et al., 2013). Expression data indicated that *OsNRX* genes are expressed in all tissues, with particularly significant differences in expression in roots, lemma, palea, embryo, and endosperm (**Figure 6**, **Supplementary Table S3**). *OsNRX1a* has the highest expression level in roots, with expression decreasing as roots, embryos, and endosperm develop, while increasing as the length of the glumes (lemma and palea) increases. The expression level of *OsNRX1b* increases with the development of the endosperm. In other tissues, the expression pattern of *OsNRX1b* is similar to that of *OsNRX1a*. *OsNRX2* has the highest expression level in endosperm, with expression increasing as roots, glumes, embryos, and endosperm develop. The expression patterns of *OsNRX1a*, *OsNRX1b*, and *OsNRX2* in different tissues are complementary, and whether there is interaction between *OsNRX* gene families needs further study.

### Expression patterns of the *OsNRX* family under drought stress

Studies have indicated that the expression of the *NRX* family increases in response to drought stress in plants such as wheat, sorghum, foxtail millet (Zhang et al., 2021; Jedmowski et al., 2014; Zhang et al., 2022). These findings suggest that the *NRX* gene family may play a significant role in plants’ responses to drought stress. In this study, by analyzing the cis-acting elements of the *OsNRX* gene family, we found that the *OsNRX* family contains abscisic acid-responsive elements (ABRE) associated with drought response. The expression of OsNRX1a and OsNRX1b in rice roots was significantly higher than that in other tissues or organs. Additionally, *OsNRX1b*, *OsNRX2*, and *OsNRX3* were found to have MYB binding sites (MBS) and CCAAT-box involved in drought induction capabilities. Therefore, we hypothesize that the *NRX* family in rice also responds to drought stress.

**Figure 7:**
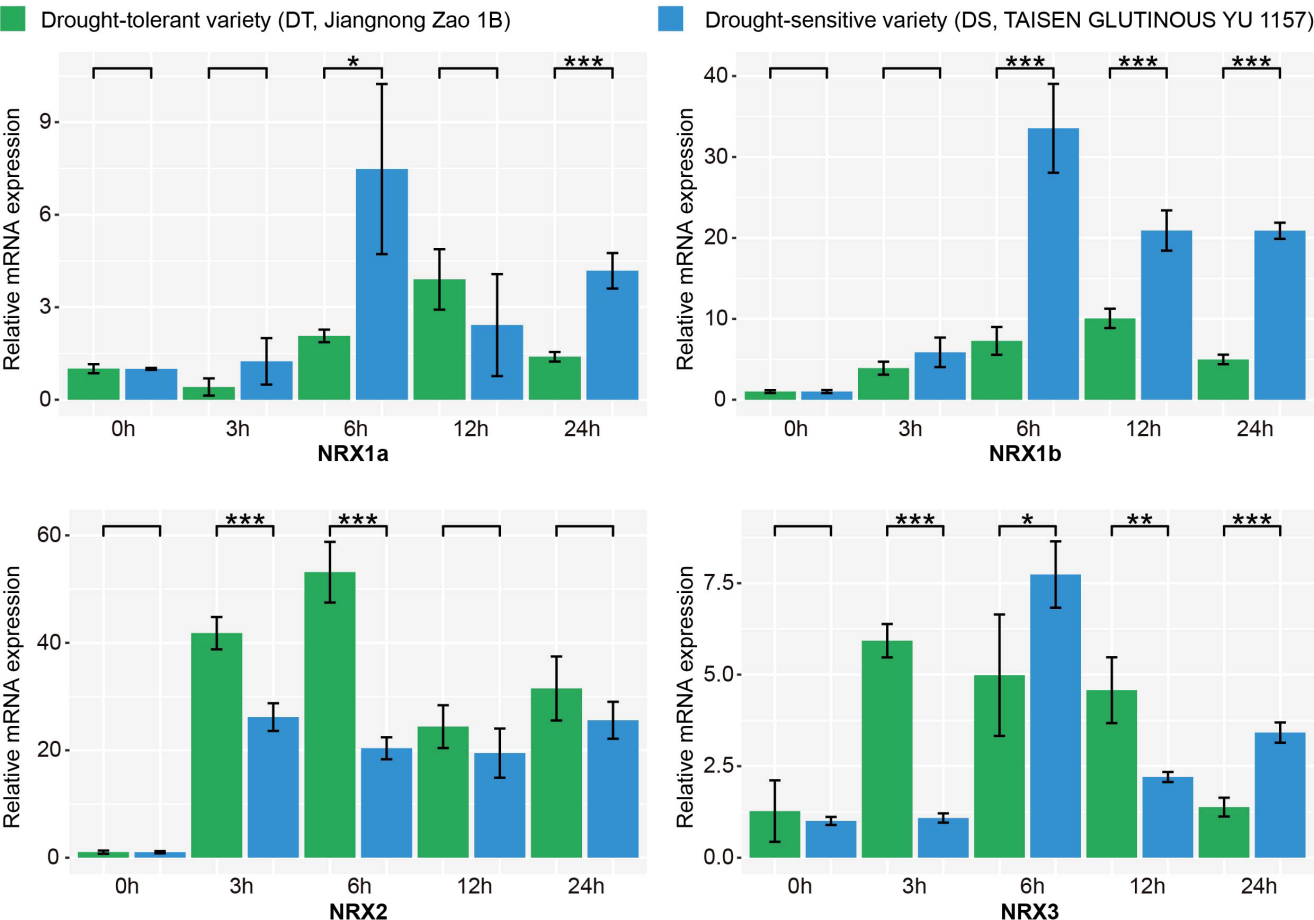
Expression changes of OsNRX gene family under PEG6000 treatment in Drought-tolerant and Drought-sensitive varieties. Response of drought-tolerant (DT, green) and drought-sensitive (DS, blue) varieties to PEG6000 treatment over time (0, 3, 6, 12, and 24 hours). Asterisks (*) indicate significant differences between DT and DS at each time point, as determined by Student’s t-test: *p < 0.1, **p < 0.05, and ***p < 0.01. The results were the mean of three independent biological replicates using quantitative real-time PCR (qRT-PCR) technology.

To validate whether the expression of the *OsNRX* family is associated with drought stress, two rice varieties, *Jiangnong Zao 1B* (drought-tolerant, DT) and *TAISEN GLUTINOUS YU 1157* (drought-sensitive, DS), were selected for qRT-PCR analysis. There were significant differences in drought tolerance between DT and DS. DT exhibited partial leaf rolling after 24 h of drought stress and nearly full recovery after rehydration, while DS showed complete leaf rolling after 24 h and no recovery after rehydration (**Supplementary Figure S1**). The gene expression level at 0h after PEG6000 treatment was used as a control, and the expression levels of *OsNRX* genes at different time points under drought treatment were analyzed (**Figure 7**, **Supplementary Table S4**). The relative expression level of *OsNRX1a* exhibited the most substantial variation in DS, with a 7.5-fold increase at 6h compared to 0h, and significant differences between DT and DS were observed at 6h and 24h. Similarly, *OsNRX1b* displayed a significant change in DS, with a 20-fold increase at 6h compared to 0h after PEG6000 treatment, and a notable difference in expression levels between DT and DS. For *OsNRX2*, the relative expression level increased by more than 20-fold after 3h of PEG6000 treatment compared to untreated samples. Specifically, DS showed a 26.1-fold increase at 3h, while DT reached a 53.2-fold increase at 6h, with significant differences in relative expression levels between DT and DS at both 3h and 6h. For *OsNRX3*, the relative expression level in DT increased 7.7-fold after 3h of PEG6000 treatment and then gradually decreased, whereas in DS, the expression level increased 7-fold after 6h, followed by a decrease and then an increase. Significant differences in relative expression levels between DT and DS were observed after 3h of treatment. In conclusion, the *OsNRX* gene family exhibited significant expression changes under drought stress, typically showing an initial increase followed by a decrease. The expression levels peaked around 6h post-stress, then decreased, but remained higher than those before the stress. Moreover, there were significant differences in the expression levels of the *OsNRX* gene family between DT and DS.

## Discussion

### Identification and characterization of the *OsNRX* gene family

The *NRX* gene family, which contains TRX-like domains, is classified within the *TRX* family. Research on the *NRX* gene family primarily stems from studies related to the *TRX* family. Zhou et al. (2022) identified 48 TRX genes in wheat, including 3 *NRX* gene family members; similarly, Zhang et al. (2022) identified 25 TRX gene family members in foxtail millet, with 3 being *NRX* gene family members. In this study, we used bioinformatics methods to identify 10 *NRX* family members in the genomes of three rice varieties (*Minghui 63*, *Nipponbare*, and *9311*). These members can be divided into three subgroups: *NRX1*, *NRX2*, and *NRX3*, and each variety contains *NRX1a* and *NRX1b* members. The physicochemical property analysis of the *OsNRX* gene family proteins revealed that these proteins are acidic and hydrophilic. In rice, the subcellular localization of both *OsNRX1* and *OsNRX2* genes includes the nucleus, which is consistent with the initial discovery of *NRX* in the nucleus (Kurooka et al., 1997; Laughner et al., 1998). However, in different rice varieties, the *OsNRX* gene family is also localized in various cellular structures. According to the study by Mata-Pérez and Spoel (2019), biotic and abiotic stresses recruit members of the *TRX* family to specific sites, which may be related to the differences in subcellular localization of *NRX* in various rice varieties. In *Minghui 63*, *Nipponbare*, and *9311*, the protein structures and conserved motifs of *OsNRX1a*, *OsNRX1b*, and *OsNRX2* are similar, which may imply similar functions. Phylogenetic tree analysis indicates that the *NRX* gene family is highly conserved in plants, particularly *NRX1*, with at least one *NRX1* gene present in every plant species.

### Impact of drought stress on *OsNRX* gene family expression

ROS are byproducts of aerobic metabolism and act as secondary messengers under various environmental stresses. When plants experience abiotic stresses such as drought, heavy metals, and salinity, excessive ROS are produced, causing plant cell damage and even cell death (Gill and Tuteja, 2010; Panda et al., 2021). To maintain the dynamic balance of ROS, plants rely on antioxidant defense mechanisms, which are activated by antioxidant enzymes such as catalase (CAT) and superoxide dismutase (SOD), as well as non-enzymatic antioxidant components (Das and Roychoudhury, 2014). CAT requires interaction with its PEX5 site on the peroxisomal membrane to enter the peroxisome and eliminate ROS. However, excessive ROS can lead to mono-ubiquitination of PEX5, preventing CAT from maintaining its reduced state and entering the peroxisome. *NRX1* can bind to CAT, maintaining its reduced state and thus protecting plant cells from oxidative stress induced by adverse environmental conditions (Kneeshaw et al., 2017; Calderón et al., 2018). Davoudi et al. (2022) found that under drought stress, the expression of *NRX* and CAT in grafted cucumber and pumpkin significantly increased, enhancing resistance to drought stress. In foxtail millet, Zhang et al. (2022) constructed transgenic *Arabidopsis* plants expressing *SiNRX1*, and the results showed that the overexpression lines had significantly higher antioxidant capacity and ROS scavenging ability under drought stress than the wild type, enhancing their drought and salt tolerance.

By analyzing the cis-acting elements of the *OsNRX* family, we found that the *OsNRX* family contains a large number of elements associated with drought stress, particularly the abscisic acid-responsive element (ABRE), which accounts for about 45% of all uncommon cis-acting elements. Abscisic acid is an important signaling hormone in plants’ response to drought stress, and the ABRE cis-acting element is a key component in the abscisic acid signaling pathway. Cheng et al. (2021b) studied the promoter of *TaNRX1* and found that a 36 bp fragment containing two ABRE cis-acting elements is a key sequence for *TaNRX1* gene response to polyethylene glycol (PEG) and abscisic acid (ABA). In plants, PEG is commonly used to simulate drought stress because it can induce physiological responses similar to drought conditions by reducing osmotic potential (Pereira et al., 2020; Fan et al., 2021; Wang et al., 2022). ABA, as an important hormone regulating plant stress resistance, can enhance plants’ tolerance to drought stress by inducing the expression of a series of stress-related genes (Liu et al., 2022). The expression patterns of the *OsNRX* family at different developmental stages indicate that *OsNRX1a* and *OsNRX1b* have significantly higher expression in rice roots than in other tissues or organs, and as the roots develop, the expression levels of *OsNRX1a* and *OsNRX1b* gradually decrease, while the expression level of *OsNRX2* gradually increases. Therefore, we speculate that the function of the *OsNRX* gene family is related to drought stress. To verify this hypothesis, we conducted drought simulation stress on two rice varieties with different seedling drought resistance. The results showed that the expression of the *OsNRX* gene family changed significantly, and the expression patterns were not the same in rice varieties with different drought resistance capabilities, indicating that the *OsNRX* family may have a regulatory function in drought resistance in rice.

SNP analysis of the OsNRX gene family (**Supplementary Table S7**) revealed one SNP in NRX1a and five SNPs in NRX2 among Nipponbare, Minghui63, 9311, Jiangnong Zao 1B, and TAISEN GLUTINOUS YU 1157, while no SNPs were detected in NRX1b or NRX3. Integrated with drought-responsive expression patterns, NRX2 exhibited significant upregulation at 3 h post-stress and differential expression between drought-tolerant (DT) and drought-sensitive (DS) varieties (**Figure 7**), potentially influenced by genetic variation. NRX1a showed differential expression between DT and DS at 24 h post-stress, likely due to its single SNP. Despite the absence of SNPs, NRX1b was rapidly upregulated at 6 h and exhibited differential expression between DT and DS, possibly regulated by upstream transcription factors or epigenetic modifications. Similarly, NRX3, despite lacking SNPs, showed differential expression between DT and DS, potentially mediated by post-transcriptional or epigenetic regulation. These hypotheses require further experimental validation.

### Potential of *OsNRX* gene family for drought resistance breeding

Among the *OsNRX* gene family, *NRX2* and *NRX1b* emerge as the most promising candidates for drought resistance breeding. *NRX2* exhibited the most dramatic response to drought stress, with relative expression levels increasing by over 20-fold within 3-12h post-PEG6000 treatment (**Figure 7**, **Supplementary Table S4**). Notably, the drought-tolerant (DT) variety showed a 53.2-fold increase at 6 h, significantly higher than the drought-sensitive (DS) variety (26.1-fold at 3 h), suggesting its critical role in rapid drought adaptation. 5 SNPs within the *NRX2* between DT and DS varieties may underlie the observed expression differences and contribute to drought tolerance. Despite lacking SNPs, NRX1b displayed a 20-fold upregulation in DS at 6 hours post-stress, with significant differences between DT and DS (**Figure 7**). Studies in wheat and foxtail millet have demonstrated that *NRX1* homologs (*TaNRX1*, *SiNRX1*) directly enhance drought tolerance by modulating antioxidant pathways and stress-related gene networks (Zhang et al., 2022; Zhou et al., 2022).

Although *NRX1a* showed a 7.5-fold increase in DS at 6 h and *NRX3* showed a 7-fold increase in DS at 6 h, the magnitude of change in their relative expression in the wake of drought stress was not as large as that of *NRX2* and *NRX1b*. The strong drought-inducible expression of *NRX2* and *NRX1b*, coupled with their genetic/epigenetic plasticity, positions them as prime targets for marker-assisted selection or CRISPR-based editing to enhance drought tolerance. Future studies should prioritize functional characterization of *NRX2* and *NRX1b* to unravel their mechanistic roles and translational potential.

### *OsNRX* gene family may regulate rice grain size

In addition to environmental influences, the size of plant seeds depends on the genotype of the embryo and endosperm and is limited by the pericarp (Li and Li, 2016). In *Arabidopsis*, Wang et al. (2010) found that the *IKU1* mutant leads to reduced endosperm growth and smaller grain size; *IKU1* is expressed in early endosperm, and restoring *IKU1* function in the endosperm can increase seed size. In rice, Ren et al. (2018) knocked out *fzp* and found that rice cell proliferation and expansion were affected, the pericarp was smaller than the wild type, and ultimately the rice grain size was reduced. By analyzing the expression patterns of *OsNRX* at different developmental stages, we found that as the length of the pericarp increases, the expression level of *OsNRX* also tends to rise. As the embryo and endosperm develop, the expression level of *OsNRX1a* gradually decreases, while the expression level of *OsNRX2* gradually increases, and the expression level of *OsNRX1b* changes little. Therefore, we speculate that the *OsNRX* gene family may have a function in regulating the size of rice grains in rice, which needs further verification by experiments.

## Conclusion

Using bioinformatics approaches, we identified a total of 10 *OsNRX* genes across three rice varieties, with *Minghui63* harboring 3, *Nipponbare* 3, and *9311* 4. All identified *OsNRX* proteins are hydrophilic. The expression of *OsNRX* genes is influenced by drought stress and varies among rice varieties with differing drought resistance, suggesting a potential role for *OsNRX* in regulating drought tolerance. The expression levels of *OsNRX* change with the development of the glumes, and the presence of cis-acting elements related to growth and development within 2000 bp upstream of their promoters, which suggests that *OsNRX* may be involved in regulating rice grain size.

## Data Availability

Genomic data and annotation files for the rice varieties *Nipponbare* and *9311* were downloaded from the Ensembl Plants database (http://plants.ensembl.org/info/data/ftp/index.html) (Yates et al., 2022), accessed on 2023.7.27. The whole-genome data and its annotation files for the rice variety Minghui63 were obtained from the Rice Information Network RIGW (http://rice.hzau.edu.cn/cgi-bin/rice_rs1/download_ext) (Song et al., 2018), accessed on 2023.7.27. The HMM file for the NRX gene (PF13905) was downloaded from the InterPro database (https://www.ebi.ac.uk/interpro) (Paysan-Lafosse et al., 2023), accessed on 2023.7.27. The genotype data used for SNP Analysis were obtained from Meng et al. (2022).
