# Supplementary material for: Genome-wide identification of the nuclear redox protein gene family revealed its potential role in drought stress tolerance in rice
Source: Front Plant Sci. 2025 Apr 22;16:1562718. doi: 10.3389/fpls.2025.1562718 (PMC12052764; doi:10.3389/fpls.2025.1562718)
Supplement: Supplementary file 1 [file DataSheet1.zip › Fig S1.Effect of stress on drought-tolerant (DT) and drought-sensitive (DS) varieties..pdf]

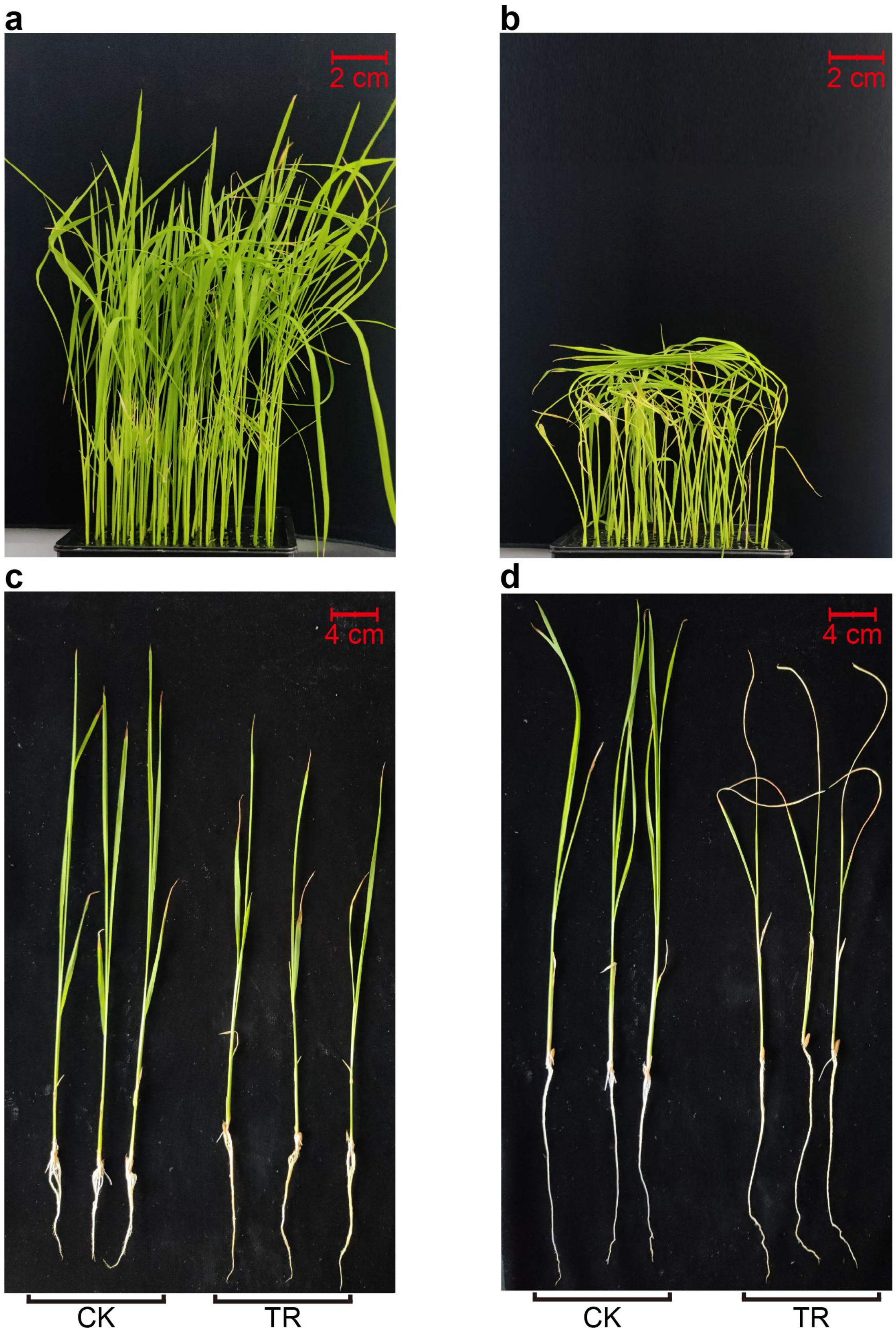

Effect of stress on drought-tolerant (DT) and drought-sensitive (DS) varieties. a.DT after 24h of PEG6000 treatment; b.DS after 24h of PEG6000 treatment; c.DT after 12 d of continued incubation; d.DS after 12 d of continued incubation. TR. 9 d of PEG6000 treatment followed by restoration of full-strength Yoshida nutrient solution incubated for 3d; CK.control group not treated with PEG6000 and incubated with full-strength Yoshida nutrient solution for 12d.
